# Assessing Self-Efficacy and Communication Regarding Sexual Agreements among Men Who Have Sex with Men in the USA: Development and Validation of Two Novel Scales

**DOI:** 10.3390/ijerph18189727

**Published:** 2021-09-15

**Authors:** Torsten B. Neilands, Deepalika Chakravarty, Lynae A. Darbes, Nathan P. O’Brien, Ilse S. Gonzalez, Colleen C. Hoff

**Affiliations:** 1 Center for AIDS Prevention Studies, Department of Medicine, Division of Prevention Sciences, University of California San Francisco, San Francisco, CA 94158, USA; deepc@ucsf.edu; 2 Center for Sexuality and Health Disparities, Department of Health Behavior and Biological Sciences, School of Nursing, University of Michigan, Ann Arbor, MI 48104, USA; lynaed@umich.edu; 3 Center for Research and Education on Gender and Sexuality, San Francisco State University, San Francisco, CA 94103, USA; natepobrien@gmail.com (N.P.O.); igonzalez4@sfsu.edu (I.S.G.); choff@sfsu.edu (C.C.H.)

**Keywords:** MSM, self-efficacy, communication, gay couples, sexual agreements, HIV prevention

## Abstract

HIV disproportionately impacts men who have sex with men (MSM) in the USA. Building upon research on relationship constructs unique to MSM couples’ HIV-prevention needs, we developed two new scales measuring sexual agreement self-efficacy (SASE) and importance of sexual agreement communication (ISAC). Following qualitative item development, we used two large independent samples of MSM couples (N1 = 441, N2 = 388) to conduct scale validation. Exploratory factor analyses indicated both SASE and ISAC to be unidimensional with 7 and 5 items (eigenvalues = 5.68 and 3.50), respectively, with strong factor loadings. Confirmatory factor analyses yielded satisfactory model fit for SASE (CFI = 0.99; SRMR = 0.03) and ISAC (CFI = 0.99; SRMR = 0.05). Reliability was high for SASE (ω = 0.92) and ISAC (ω = 0.84). Predictive validity analysis revealed a protective association between higher scores on both scales and the outcomes of sexual risk behavior and agreement breaks. Convergent and discriminant validity analyses demonstrated associations in the expected directions between these scales and multiple measures of relationship quality. Therefore, SASE and ISAC are two brief, valid, and reliable scales that can facilitate more in-depth explorations of sexual agreements in MSM and thereby contribute greatly to improving our understanding of and ability to intervene on sexual agreements to improve health and relationship outcomes.

## 1. Introduction

Men who have sex with men (MSM) continue to represent a disproportionate percentage of individuals impacted by HIV in the USA [1]. Most MSM will have a primary male partner during their lives at some point. In fact, studies show that over half of MSM report they are in a committed relationship with another man [2,3,4]. Past epidemiological reports of HIV transmission found that up to 68% of new infections occurred in the context of a committed relationship [5]. Although research with male couples has received much needed attention recently [6,7], a dearth of research regarding key aspects of MSM relationships, such as sexual agreements, remains. Specifically, there are few measures available to assess the unique aspects of male couples’ sexual agreements. This gap is more striking given that the majority of male couples report having a sexual agreement about whether sex with partners outside of the relationship is permitted [4,8,9]. Couples who have agreements where sex with outside partners is permitted (e.g., non-monogamous agreements) must negotiate the parameters of acceptable behaviors. For example, some couples allow sex with outside partners only if a condom is used while others do not allow anal sex at all with outside partners. Some couples allow outside sex only when a partner is traveling and others may allow it anytime but have parameters about who the outside partner is and where the outside sexual encounter occurs. Other couples prefer to have sex with outside partners together at sex parties or in three-way sexual encounters. Couples who have agreements where sex with outside partners is not permitted (e.g., monogamous agreements) may have fewer parameters to negotiate but may be more vulnerable to broken agreements, which could potentially put the couple at risk for HIV [10]. Specifically, if an HIV-negative partner has condomless anal sex (CAS) with an outside partner who is HIV-positive or of unknown HIV status and then engages in CAS with his HIV-negative primary partner, both partners in the primary relationship could be at risk for HIV transmission [11] if the outside partner was HIV-positive with a detectable viral load, and neither of the HIV-negative partners were taking pre-exposure prophylaxis (PrEP) medications.

In recent years, HIV-prevention research has identified unique prevention needs for male couples across the world [2,11,12,13,14]. For example, positive relationship factors, such as satisfaction, trust, and commitment, are associated with less sexual risk with outside partners [11,15]. Additionally, couples who report that they are satisfied with their agreements are also less likely to be at risk for HIV [16]. Couples who have the same understanding of what their agreement is report greater relationship satisfaction and less HIV risk [17]. Further, it is important to not only determine the content of sexual agreements but also to recognize that aspects of sexual agreements, such as satisfaction with and value of those agreements, represent components of relationship quality akin to levels of commitment or trust [18].

Motivations for having sexual agreements are important to understand so that prevention efforts can support couples in having satisfying agreements and relationships [2]. These motivations include a desire to prevent HIV transmission, increase sexual satisfaction, wanting an emotionally satisfying relationship, and having appropriate structure and boundaries in the relationship [9]. Previous research has shown that the more invested a partner is in his agreement, the more satisfied he is in his relationship as well as being at less risk for HIV infection [19]. This is key because if a partner is more invested in his agreement, he is more likely to adhere to the agreement and avoid broken agreement scenarios and their accompanying potential for HIV risk. Recognizing the importance of agreement investment, researchers, including our team [19], have developed instruments to measure men’s investment in their sexual agreements. However, unexplored in this context are additional factors aside from investment that would be amenable to changes through interventions that could increase men’s adherence to their agreements, which could in turn result in better sexual health. Two such mechanisms of action are communication and self-efficacy.

Communication is a key component of relationships. Good communication is associated with greater relationship satisfaction and lower rates of divorce [20]. Conflict-resolution skills have also been associated with greater relationship satisfaction in the early stage of relationships [21]. However, little is known about how communication contributes to successful agreement negotiation. How couples feel about the conversations they have about their agreement as well as the skills that they bring to those conversations could be a key factor in their satisfaction with their agreement, their adherence to the agreement and, ultimately, the success of their agreement.

Self-efficacy has also been an important construct in HIV prevention over the course of the epidemic but has been focused on individual-level behavior and has not been examined in the context of intimate relationships or sexual agreements. Theories such as the AIDS Risk Reduction Model and the Health Belief Model [22,23] posit the importance of self-efficacy in behavior change for individuals but do not address self-efficacy in the context of sexual agreements. It is not yet known how self-efficacy specifically regarding sexual agreements supports agreement negotiations or adherence to agreements. Thus, it is not clear how much of the success of an agreement can be attributed to communication skills versus how efficacious one feels about honoring and adhering to his agreement.

While sexual agreements are ubiquitous and crucial for the sexual health of male couples, to date, there are few quantitative scales [19] available to measure aspects of sexual agreements and none that specifically measure self-efficacy or communication regarding sexual agreements. The present work aimed to build on previous scale development research that measures relationship constructs unique to gay male couples’ HIV-prevention needs [19]. Here, we describe the development of two independent scales focusing on the distinct entities of sexual agreement self-efficacy (SASE) and importance of sexual agreement communication (ISAC) that researchers can use either singly or together depending on their research questions.

## 2. Methods

The scale development process consisted of three distinct phases, each from its unique independent study of gay couples. The first consisted of qualitative interviews that formed the basis of the items for the scales, while the second and third phases were to collect quantitative data to facilitate validation of the scales. The first phase has been described in detail previously [19]; information about that phase included in the present paper is meant to provide an overview only. The present paper focuses in-depth on analyses of data from the two subsequent quantitative studies.

Samples: For the qualitative phase of item development, 39 gay couples were recruited in the San Francisco Bay Area. For the present quantitative analyses, data from two large independent studies of gay male couples were used. The first sample (*N* = 441 couples; hereafter referred to as Study 1) was recruited between February 2012 and August 2013 in the Greater San Francisco Bay area. The second sample (hereafter referred to as Study 2) was recruited simultaneously in the Greater San Francisco and New York City metropolitan areas in two independent and non-overlapping phases from June 2012 to May 2013 (*N* = 171 couples) and from August 2013 to October 2014 (*N* = 217 couples) for a total of 388 couples.

Recruitment: For all three studies, the couples were recruited from community venues, such as street fairs, bars, community centers, churches, and local businesses using active and passive recruitment strategies that included distributing study postcards, posting study flyers, and placing advertisements inviting interested potential participants to call a toll-free recruitment hotline for information. For Study 2, social media platforms, such as Facebook and Grindr, were also used for recruitment. Callers and their partners were screened individually, and each partner had to meet the eligibility criteria for the couple to be eligible for participation.

Eligibility criteria: The common eligibility criteria for all three studies were as follows: each participant had to be at least 18 years old, know their own and partner’s HIV status, be fluent in English, and not identify as transgender. At least one of the partners had to have engaged in anal sex in the previous three months and couples had to be either seroconcordant HIV-negative (i.e., both partners are HIV-negative) or serodiscordant (i.e., one partner is HIV-negative and the other is HIV-positive). Additional eligibility criteria that differed by study are outlined below.

Qualitative interviews and Study 1: Couples had to have been in the relationship for at least three months.

Study 2: Each participant had to report their primary racial identity as Black or White and had to have lived in the U.S. since age 7 years or younger. Couples had to have been in the relationship for at least six months.

Procedures: Eligible couples were scheduled for an in-person visit to the study offices in downtown San Francisco (as well as New York City, for Study 2), where informed consent was obtained from each participant prior to participation. To provide privacy and encourage independent responses, the interviews were conducted by trained interviewers simultaneously but separately to allow each partner to speak independently and freely. Interviews were recorded and then transcribed verbatim. Similarly, the surveys were administered simultaneously but separately via audio computer assisted self-interview (A-CASI). The interviews and the surveys covered topics such as relationship dynamics, sexual risk behavior, and sexual agreements. Participants were provided a cash incentive of $40 to compensate them for their time and contribution. All study procedures were reviewed and approved by the Institutional Review Boards of University of California, San Francisco (for the qualitative phase), San Francisco State University (for Studies 1 and 2), and Columbia University (for Study 2).

Item development: Qualitative interviews conducted to investigate sexual agreements and factors surrounding the maintenance of those agreements yielded the potential pool of items for the SASE and ISAC scales. The analysis of the interviews was guided by grounded theory [24,25], which allowed thematic categories to emerge from the data. Codes were generated by study staff and included agreements concerning sexual activity within the relationship, agreements concerning sexual activity outside the relationship, sexual behaviors within the relationship, sexual behaviors outside the relationship, perceptions of risk, gay identity, protective factors (e.g., actions or beliefs regarding the protection of the relationship, the individual or partner, or sexual safety), relationship dynamics, and other (e.g., coded text that did not fall into any other category). Within those categories, scale items were generated from codes that focused on agreements and relationship dynamics. Agreement codes included agreement type (including those that were monogamous, allowed sex with outside partners, and allowed threesomes), agreement motivation, maintenance of or commitment to the agreement, agreement acceptability, the explicitness or implicitness of the agreement, agreement change, broken agreements, and disclosure of broken agreements. Codes that focused on relationship dynamics included satisfaction, honesty, trust, intimacy, couple serostatus, and motivation. Once codes were identified, staff applied them to selected sections of the transcripts to verify code definition and coding consistency within the team. When agreement was found among research staff, the transcripts were coded. The coding process began with two study staff members (coders) coding the same transcript independently of one another. Afterwards, they met to compare their coded transcripts for discrepancies. With a third staff member, coders reconciled any discrepancies. This process was repeated until both coders demonstrated consistent coding techniques (approximately the first 10 transcripts coded). All subsequent interviews were coded independently by one of two coders and verified by a third staff member [26].

Following the completion of coding, we employed content analysis to identify the primary categories that address agreement communication and one’s ability to maintain his agreement [27]. Survey items were generated for each of these categories (12–15 items per category) and reviewed by the study team, who then made modifications to the items to enhance clarity and minimize redundancy. As a final step, the study team conducted a series of cognitive interviews with volunteers to ensure item reliability [28,29]. Three members of the study team, trained in cognitive interviewing, queried the volunteers to obtain details regarding how they arrived at their responses to each item. Questions in the cognitive interviews included “What did the question mean to you?”; “Did you understand what we were looking for in that question?”; and “Was there anything in that question that was confusing?” Responses were recorded, summarized, and reviewed by the study team. The cognitive interview and review process further honed the items, resulting in the set of questions that formed the potential pool for the SASE and ISAC scales. These items (10 for SASE, 7 for ISAC) were administered to the participants in the surveys in two subsequent independent studies described below.

Measures in the quantitative studies:

Sample characteristics: Participants reported their age, relationship length, education, employment, income, race, and HIV status.

Relationship measures: Data collected for a number of relationship measures (Table 1) were used to demonstrate the convergent and discriminant validity of the SASE and ISAC scales.

Sexual risk: Participants reported in depth about their sexual interactions in the past three months with outside partners of concordant, discordant, and unknown HIV status. These responses in conjunction with the participant’s own HIV status were used to determine whether the participant had CAS with an outside partner of discordant or unknown serostatus in the past three months (0 = No; 1 = Yes).

Sexual agreements: Responses from both partners were used to obtain the couples’ agreement type: monogamous (the agreement is to have sex only with each other) and non-monogamous (partners have either agreed to allow sex with outside partners, with or without conditions; or one partner reported the agreement as monogamous while the other reported it as non-monogamous). Participants also reported whether they ever broke their current agreement (0 = No; 1 = Yes).

Candidate items for the SASE and ISAC scales: These are a total of 17 questions (10 for SASE and 7 for ISAC) that form the basis of the present validation analyses.. Each question had five-point Likert-type response options: Not at all, A little, Moderately, Very much, Extremely [36].

Data Analyses: SAS software (Version 9.4 for Windows, SAS Institute Inc., Cary, NC, USA) was used to compute proportions, means, and standard deviations to characterize each sample.

Exploratory factor analysis (EFA) using data from Study 1: Next, separate EFAs were performed on the pool of self-efficacy and communication items using FACTOR 10 [37]. The adequacy [38,39] of the correlation matrices for both sets of items were examined using the matrix determinant (>0.00001), Bartlett’s sphericity statistic (*p* < 0.05), and the Kaiser–Meyer–Olkin (KMO) test (>0.5). The Hull method [40] was used to determine the number of factors to retain for each scale. Items’ uni-dimensionality was assessed via the explained common variance (I-ECV) and mean of residual absolute loadings (I-REAL) [41]. I-ECV values of 0.85 or larger and I-REAL values of 0.30 or lower support treating an item as being a measure of a unidimensional underlying latent factor. Because our goal was to develop unidimensional measures of each latent factor, I-ECV and I-REAL were used to identify individual non-unidimensional items and drop them from further consideration.

Confirmatory factor analysis (CFA) using data from Study 2: Next, CFAs were performed on the items and factor structure obtained from the EFAs using M*plus* version 8 (Muthén & Muthén, Inc., Los Angeles, CA, USA) [42]. Due to the items’ ordinal responses, a diagonally weighted least-squares estimator (M*plus* estimator WLSMV) was used [43]. Global model fit was assessed using the chi-square test of exact fit. Due to the chi-square test’s rejection of models resulting from trivial amounts of lack of fit at large N, we also evaluated approximate model fit. Specifically, we followed Hu and Bentler’s recommendation [44] of a two-index strategy of using Standardized Root Mean Square Residual (SRMR ≤ 0.08) supplemented with one of multiple statistics, including Comparative Fit Index (CFI ≥ 0.95) and Root Mean Squared Error of Approximation (RMSEA ≤ 0.06). We based our decision on SRMR and CFI due to RMSEA’s positive bias in low degree-of-freedom models of the type fitted in this paper [45] while reporting all three indices for completeness. Internal consistency reliability was estimated using McDonald’s omega (ω).

Predictive validity using data from both studies: These analyses employed generalized estimating equations (GEEs) fitted by SAS PROC GENMOD to examine the bivariate associations of the two new scales with two behavioral sexual risk variables: (a) any CAS with an outside partner of discordant or unknown HIV serostatus and (b) whether the participant ever broke his sexual agreement. Given the binary nature of these two outcomes, GEEs used a binary distribution and logit link, yielding odds ratios per unit change in scale scores. We hypothesized that higher levels of sexual agreement self-efficacy and importance assigned to communication regarding sexual agreements would be associated with lower odds of broken agreements and of CAS with outside partners of discordant or unknown HIV status. GEEs were fitted assuming an exchangeable correlation structure to account for clustering of individual participants within couples.

Convergent and discriminant validity using data from Study 2: To assess these, we correlated the two new scales with the relationship measures shown in Table 1. To account for the clustering of individual participants within couples, we computed these correlations via full-information maximum likelihood (FIML) with 95% confidence intervals and test statistics supplied by Yuan and Bentler’s cluster-adjusted heteroskedastic-consistent non-normality-corrected estimator T_2_* [46] via the MLR estimator in M*plus*. We hypothesized that scores on the SASE and ISAC scales would be positively correlated with sexual agreement investment, relationship satisfaction, commitment, mutual constructive communication, trust, and internal control. Similarly, we expected that the scores on these new scales would be negatively correlated with perceived quality of relationship alternatives and mutual avoidance and withholding. Further, we expected no meaningful association between these new measures and alcohol abuse.

## 3. Results

Participant characteristics: Study 1’s sample was racially diverse, with 61% White, 13% Hispanic, 10% Asian/Pacific Islander, 8% Black, and 8% mixed-race participants, whereas Study 2’s sample, by the design of the primary study, consisted of 65% White and 35% Black men (Table 2). Participants’ non-race characteristics in the two samples were generally comparable with Study 1’s sample being marginally older (41 vs. 38 years), with longer relationship lengths on average (8 vs. 6 years), higher educational attainment (59% with at least a Bachelor’s degree vs. 48%), lower unemployment (26% vs. 28%), and higher incomes (36% with incomes over $60,000 vs. 26%) compared with Study 2’s sample. Conversely, Study 1’s sample reported higher rates of broken agreements (30% vs. 22%) and CAS with outside partners of discordant or unknown HIV status in the previous three months (12% vs. 10%). In both samples, approximately three quarters of the couples were seroconcordant HIV-negative and a quarter were serodiscordant; approximately 42% reported monogamous agreements.

Exploratory Factor Analyses using data from Study 1: The EFA for the SASE scale started with ten candidate items. The statistics evaluating the adequacy of the correlation matrix were satisfactory (Table 3). The Hull method indicated support for a single common factor. Uni-dimensionality screening indicated that three of the items did not contribute satisfactorily to uni-dimensionality: “When you are under the influence of drugs or alcohol, how difficult is it for you to honor your current agreement?” (I-ECV = 0.24; I-REAL = 0.49); “When you see friends breaking their agreements, how difficult is it for you to honor your current agreement?” (I-ECV = 0.23; I-REAL = 0.87); and “When you see other gay men breaking their agreements, how difficult is it for you to honor your current agreement?” (I-ECV = 0.30; I-REAL = 0.71). These three items were therefore dropped from further consideration. Target I-ECV and I-REAL thresholds were achieved or exceeded for the remaining seven items whose factor loadings were also strong, achieving a magnitude of |0.60| or larger and were retained.

For the ISAC scale, the EFA began with seven items. The statistics evaluating the adequacy of the correlation matrix were satisfactory (Table 3). The Hull method supported the presence of a single common factor. Only two items did not achieve target uni-dimensionality thresholds: “How difficult is it to talk to your primary partner about your current agreement?” (I-ECV = 0.23; I-REAL = 0.74); and “How fearful are you about talking to your primary partner about your current agreement?” (I-ECV = 0.21; I-REAL = 0.76). These two items were therefore dropped from further consideration. The remaining five items met or exceeded the target uni-dimensionality thresholds and demonstrated strong factor loadings of |0.69| or larger and were retained.

Confirmatory factor analyses using data from Study 2: CFAs of the final factor structures implied by the EFAs from Study 1 applied to Study 2’s data yielded good fit for both the new scales: SASE (χ^2^(14) = 462.61, *p* < 0.0001; SRMR = 0.03, CFI = 0.99, RMSEA = 0.20) and ISAC (χ^2^(5) = 229.57, *p* < 0.0001; SRMR = 0.05, CFI = 0.99, RMSEA = 0.24). Factor loadings were strong and estimated with high precision (Table 3). Reliability as characterized by McDonald’s omega was high for both SASE (ω = 0.92; 95% CI: (0.90, 0.93)) and ISAC (ω = 0.84; 95% CI: (0.82, 0.86)). The resulting SASE and ISAC scales along with their scoring instructions are presented in Appendix A and Appendix B, respectively.

Predictive validity analyses using data from Study 1 and Study 2: GEEs showed a protective association between each of SASE and ISAC, and each of the two outcomes of interest: CAS with outside partners and a history of breaking one’s agreement. Specifically, higher scores on SASE and ISAC were associated with lower odds of sexual risk behavior (Table 4). These associations were generally similar in magnitude across the two studies and were stronger for self-efficacy than for communication. In fact, SASE was significantly associated with CAS with outside partners across both studies, whereas ISAC was significantly associated with CAS in Study 1 but not in Study 2. Taken collectively, these findings offer support for both new scales being associated with important behavioral measures of sexual risk, with SASE demonstrating the stronger of the two scales’ associations.

Convergent and discriminant validity using data from Study 2: In line with our hypotheses, the SASE and ISAC scales were positively and statistically significantly associated with sexual agreement investment, relationship satisfaction, commitment, mutual constructive communication, trust, and internal control (Table 5). The strongest associations were with sexual agreement investment and commitment, which is to be expected given the thematic relatedness of these constructs to the two new agreement-related scales. More moderate but still significant associations were observed with the remaining variables. Additionally, as hypothesized, both SASE and ISAC were negatively associated with quality of relationship alternatives and mutual avoidance and withholding, which are both measures of poor relationship health. As anticipated, alcohol abuse was uncorrelated with both SASE and ISAC.

## 4. Discussion

Our findings based on two large independent samples of intact gay couples living in the San Francisco Bay Area and New York City demonstrate that the SASE and ISAC scales are reliable and valid for respectively assessing the levels of sexual agreement self-efficacy and the importance attributed to communication regarding one’s sexual agreement. Both scales demonstrated satisfactory fit via confirmatory factor analysis as well as high reliability. Predictive validity analyses supported our hypotheses regarding the protective associations between both self-efficacy and communication and the two primary outcomes of interest: condomless anal sex with outside partners of discordant and unknown HIV status and breaking one’s agreement. Further, these newly developed scales were found to associate with established markers of relationship quality (e.g., commitment, trust, etc.) in the hypothesized directions. These findings align with prior research regarding the importance of determining not only the presence and type of sexual agreements but also account for their specific aspects such as investment in, communication about, and self-efficacy in maintaining them. Thus, our findings suggest that the SASE and ISAC scales represent novel additions to our conceptualization of relationship quality and underscore the crucial role of sexual agreements in the relationship context for gay men.

The findings for convergent and discriminant validity were consistent for both scales, across both samples, and were in alignment in the hypothesized directions for positive relationship qualities, including trust, commitment, satisfaction, communication, sexual agreement investment, and internal control. Previous work has posited that these aspects of sexual agreements (investment in, value of, and motivations for) should be considered to be indicators of relationship quality akin to more traditional conceptualizations of satisfaction and trust [2,18]. The current results for sexual agreement self-efficacy and communication suggest additions to the constellation of sexual agreement-related relationship factors that should be incorporated into our understanding of the relationship contexts of male couples.

Previous literature has identified that male couples may not always have an accurate shared understanding of their agreements [47]. The ability to assess the level of importance assigned to communication regarding an agreement might provide much-needed support for agreement negotiation and maintenance and a reduction in agreement discrepancy, which has been found to be negatively associated with sexual risk behavior [6]. Other efforts have tested electronic health HIV-prevention toolkits to encourage agreement formation [14]. Those interventions could also benefit from a standardized assessment of agreement communication and self-efficacy, thereby improving these novel methodologies and expanding their reach among male couples. Agreements have also been found to influence potential uptake of prevention strategies, such as PrEP [7] and couples-based HIV testing and counseling [48]. Further, our finding that scores on SASE and ISAC were significant correlates of key behavioral indicators of HIV risk, including condomless anal sex and breaking one’s agreement, underscores the importance of including them in future prevention research studies. Pragmatically, our findings suggest that these two scales could substantially enhance our ability to tailor prevention efforts for male couples to increase uptake of efficacious HIV-prevention strategies, such as PrEP.

There are multiple strengths of our study, including racially and ethnically diverse samples from two separate geographic areas (San Francisco Bay Area and New York City), the inclusion of both concordant HIV-negative and HIV-serodiscordant couples, and the inclusion of couples with both monogamous and non-monogamous sexual agreements. Our rigorous scale development process included theory- and qualitative interview-based item development and cognitive interviews to strengthen item comprehensibility followed by validation of psychometric properties using multiple large independent samples. We are unaware of other scales in the literature that measure the importance that gay men assign to communication about their sexual agreement or one’s self-efficacy in maintaining one’s sexual agreement or of studies investigating such constructs with HIV-prevention outcomes (e.g., condomless anal sex and broken agreements), suggesting a novel contribution.

Despite its many strengths, our study also has certain limitations. First, the generalizability of the findings is limited both by the non-probability nature of the samples as well as the limited metropolitan areas in the USA from which they were recruited. Future studies should investigate the performance of these scales in non-metropolitan USA and global metropolitan and non-metropolitan settings. Second, we were not able to make causal inferences due to the cross-sectional nature of the data. Third, the data for the present analyses were collected approximately ten years ago, and much has changed in relation to HIV prevention during that time. However, sexual agreements among gay male couples remain ubiquitous, and investigations regarding them continue to uncover insights that can prove valuable for sexual health and relationships [49,50,51]. Specifically, aspects of sexual agreements, such as quality and value, are akin to other relationship quality constructs, such as commitment and satisfaction [18,21]; the two new scales presented here are aligned with such aspects of relationship quality, which endure over time even in the context of the continuously evolving HIV landscape and HIV-risk literature. Thus, understanding the importance of and being able to assess levels of self-efficacy and communication about sexual agreements remain relevant for both HIV and non-HIV research contexts. Finally, although broken agreements can have consequences for relationship quality and potential HIV transmission, an in-depth investigation of the downstream sequelae of broken agreements is beyond the scope of this article.

There are several key implications from our findings. Given that MSM remain the population most impacted by HIV in the USA [1], strategies that contribute to HIV prevention and treatment efforts, including measures such as the SASE and ISAC scales, are worthy of consideration. Further, a high proportion of HIV infections among MSM stem from within primary partnerships [5,52]. Communication is frequently identified as being integral for gay male couples, especially in regards to developing and maintaining their sexual agreements [50,51]. The ISAC scale provides an option for researchers and practitioners to empirically determine a range of factors associated with how gay men in relationships communicate about their agreements. Similarly, the SASE scale provides an opportunity to determine the level of self-efficacy regarding keeping or honoring one’s agreement within a range of emotional or social contexts. In addition to its empirical benefit, identifying emotional states or situations that may render one’s agreement to be potentially vulnerable offers windows for intervention. For example, knowledge of one’s self-efficacy and communication regarding sexual agreements could improve one’s ability to be prepared for these vulnerable situations by having preemptive strategies to prevent broken agreements, which are known for their potential for sexual risk and are also associated with decreased relationship quality [8]. Sexual agreement strategies have evolved to include a range of approaches to prevent HIV transmission, both behavioral (e.g., condom use) and biomedical (e.g., PrEP, treatment-as-prevention), including in global contexts [14,16,53,54]. Taken together, the SASE and ISAC scales offer both researchers and practitioners tools that may improve their ability to intervene with male couples regarding their sexual agreements, thereby leading to improved HIV-prevention efforts and relationship quality outcomes among a population in need of tailored HIV-prevention approaches.

## 5. Conclusions

Among gay men, sexual agreements impact a variety of outcomes that are salient both for HIV prevention and for improving relationship quality. By facilitating more in-depth explorations of sexual agreements, these novel SASE and ISAC scales can contribute greatly to improving our understanding of, and ability to intervene on, sexual agreements in order to improve health and relationship outcomes.

## Figures and Tables

**Table 1:** Relationship measures.

Measure	Reference	Items	Response Scale	Sample Item
Rusbult Investment Model Scale:	[30]		9 point: “Do not agree at all” to “Agree completely”	
Satisfaction		5		“My relationship is close to ideal.”
Commitment		7		“I want our relationship to last for a very long time.”
Quality of Alternatives		5		“My needs for intimacy, companionship, etc., could easily be fulfilled in an alternative relationship.”
Internal Control Index ^@^	[31]	28	5-point: “Rarely (less than 10% of the time)” to “Usually (more than 90% of the time)”	“I ______ decide to do things on the spur of the moment.”
Communication Patterns Questionnaire:				
Mutual Constructive Communication	[32]	6	9-point: “Very unlikely” to “Very likely”	“During a discussion of a relationship problem, both of us express our feelings to each other.”
Mutual Avoidance and Withholding	[33]	3	9-point: “Very unlikely” to “Very likely”	“When some problem in the relationship arises, both of us avoid discussing the problem.”
Alcohol dependence ^@^	[34]	4	Yes/No	“Have you ever felt bad or guilty about your drinking?”
Sexual Agreement Investment	[19]	13	5-point: “Not at all” to “Extremely”	“How much does your current agreement matter to you?”
Trust	[35]	8	7-point with ends and midpoint labelled: “Strongly disagree”, “Neutral”, “Strongly agree”	“I feel that I can trust my partner completely.”

Note: For all scales above, higher scores represent higher levels of the characteristic under consideration. To achieve this, appropriate items within each scale were reverse scored prior to computing the composite score. ^@^: Recorded only in Study 2.

**Table 2:** Descriptive characteristics of study participants.

Individual-Level	Study 1 (*N* = 882)		Study 2 (*N* = 776)	
Age (years)/mean (SD)	41.3	(12.4)	37.69	(12.3)
Relationship Length (years)/mean (SD)	7.8	(7.9)	5.79	(7)
	n	(%)	n	(%)
Race				
White, not of Hispanic Origin	541	(61.3)	504	(65)
Black, not of Hispanic Origin	66	(7.5)	272	(35.1)
Hispanic (Latino)	115	(13)	-	-
Asian/Pacific Islander	84	(9.5)	-	-
Mixed Race/ Other	70	(7.9)	-	-
American Indian or Alaskan Native	6	(0.7)	-	-
Education				
High School/High School Equivalent (e.g., GED test passed) or less	88	(10)	168	(21.7)
Some college/Associate Degree	271	(30.7)	234	(30.2)
Bachelor’s Degree or higher	523	(59.3)	374	(48.2)
Employment				
Employed (full-time/self-employed)	543	(61.6)	419	(54)
Employed part-time	113	(12.8)	137	(17.7)
Unemployed	226	(25.6)	220	(28.4)
Income				
Less than $30,000	319	(36.2)	375	(48.3)
$30,000–$59,999	244	(27.7)	203	(26.2)
$60,000 and higher	319	(36.1)	198	(25.6)
Ever broken current sexual agreement	266	(30.2)	173	(22.4)
Reported CAS with outside partner of discordant or unknown HIV status in the previous three months	103	(11.7)	81	(10.4)
Couple-Level:	Study 1 (*N* = 441)		Study 1 (*N* = 388)	
Couple HIV status				
Seroconcordant negative	336	(76.2)	282	(72.7)
Serodiscordant	105	(23.8)	106	(27.3)
Agreement Type				
Monogamous	182	(41.3)	164	(42.3)
Non-monogamous	259	(58.7)	224	(57.7)

Notes: CAS, condomless anal sex.

**Table 3:** Standardized Factor Loadings from Exploratory and Confirmatory Factor Analyses and Item Correlation with Total.

**Item Label**	**EFA Loading**	**Item Correlation w/Total**	**CFA Loading**	**95% CI of CFA Loading**	**Item Correlation w/Total**
**Sexual Agreement Self-Efficacy (SASE) scale**	**Study 1 (*N* = 831)**		**Study 2 (*N* = 772)**		
How confident are you that you can honor your current agreement?	−0.80	0.56	0.90	(0.88, 0.92)	0.80
When someone you are attracted to is seducing you, how confident are you that you can honor your current agreement?	−0.81	0.52	0.87	(0.85, 0.89)	0.77
When you are under the influence of drugs or alcohol, how difficult is it for you to honor your current agreement?	0.29	0.06	-	-	-
When you see friends breaking their agreements, how difficult is it for you to honor your current agreement?	0.58	0.10	-	-	-
When you are feeling bad about yourself, how likely is it that you will honor your current agreement?	−0.60	0.44	0.64	(0.60, 0.68)	0.47
When you see other gay men breaking their agreements, how difficult is it for you to honor your current agreement?	0.62	0.06	-	-	-
How easy is it for you to keep your current agreement?	−0.79	0.56	0.86	(0.83, 0.88)	0.77
When you are angry with your partner, how confident are you that you will be able to honor your current agreement?	−0.93	0.59	0.96	(0.96, 0.97)	0.83
When you are anxious about your relationship, how confident are you that you will be able to honor your current agreement?	−0.97	0.61	0.96	(0.96, 0.97)	0.83
When your relationship has conflict, how confident are you that you can honor your current agreement?	−0.96	0.62	0.97	(0.96, 0.98)	0.85
**Statistics of adequacy of the correlation matrix**					
Determinant	0.002				
Bartlett’s statistic	5071.8 (df = 45; *p* < 0.001)				
Kaiser–Meyer–Olkin (KMO) test	0.87				
**Item Label**	**EFA Loading**	**Item Correlation w/Total**	**CFA Loading**	**95% CI of CFA Loading**	**Item Correlation w/Total**
**Importance of Sexual Agreement Communication (ISAC) scale**	**Study 1 (*N* = 810)**		**Study 2 (*N* = 771)**		
How important is it to talk to your primary partner about your current agreement?	−0.72	0.57	0.75	(0.71, 0.78)	0.65
How difficult is it to talk to your primary partner about your current agreement?	0.45	-0.05	-	-	-
How fearful are you about talking to your primary partner about your current agreement?	0.44	0.001	-	-	-
How much do you benefit from talking to your primary partner about your current agreement?	−0.69	0.56	0.73	(0.69, 0.76)	0.63
How important is it to talk about your current agreement when you are unclear about what it is?	−0.94	0.54	0.94	(0.93, 0.96)	0.72
How important is it to talk about your current agreement when your primary partner is unclear about what it is?	−0.93	0.51	0.98	(0.97, 1.00)	0.74
How much do you enjoy talking to your primary partner about your current agreement?	−0.64	0.35	0.67	(0.63, 0.71)	0.58
**Statistics of adequacy of the correlation matrix**					
Determinant	0.055				
Bartlett’s statistic	2341.2 (df = 21; *p* < 0.001)				
Kaiser–Meyer–Olkin (KMO) test	0.71				

Notes: EFA factor loadings were estimated using FACTOR 10; CFA factor loadings and confidence intervals were estimated using M*plus* version 8. Item-total correlations were estimated using SAS version 9.4.

**Table 4:** Associations of SASE and ISAC with self-reported sexual risk behaviors.

Explanatory Variable	Outcome Variable	Study 1			Study 2		
		Odds Ratio	95% Confidence Interval	*p*-Value	Odds Ratio	95% Confidence Interval	*p*-Value
SASE	CASOUT	0.60	(0.47, 0.78)	<0.0001	0.61	(0.48, 0.77)	<0.0001
	EVRBRK	0.29	(0.23, 0.37)	<0.0001	0.48	(0.39, 0.59)	<0.0001
ISAC	CASOUT	0.76	(0.61, 0.94)	0.0115	0.85	(0.68, 1.06)	0.1551
	EVRBRK	0.81	(0.69, 0.94)	0.0072	0.74	(0.64, 0.86)	<0.0001

Notes: SASE, Score on Sexual Agreement Self-Efficacy scale; ISAC, Score on Importance of Sexual Agreement Communication scale; CASOUT, Condomless anal sex with an outside partner of discordant or unknown serostatus; EVRBRK, Ever broken sexual agreement with primary partner. Study 1 *N* = 882 for all analyses. Study 2 *N* = 771 for SASE analyses; *N* = 770 for ISAC analyses. Odds ratios were estimated using generalized estimating equations (GEE) to account for clustering of participants within dyads and represent the change in the odds of the outcome having occurred per unit change in the explanatory variable.

**Table 5:** Correlations (*r*) of SASE and ISAC with interpersonal relationship measures from Study 2.

Relationship Correlate	SASE			ISAC		
	*R*	95% CI	*p*-Value	*r*	95% CI	*p*-Value
Sexual Agreement Investment	0.76	(0.71, 0.81)	<0.001	0.61	(0.56, 0.67)	<0.001
Relationship Satisfaction	0.42	(0.34, 0.50)	<0.001	0.32	(0.25, 0.40)	<0.001
Commitment	0.48	(0.40, 0.56)	<0.001	0.31	(0.25, 0.38)	<0.001
Quality of Relationship Alternatives	−0.30	(−0.37, −0.23)	<0.001	−0.17	(−0.24, −0.10)	<0.001
Mutual Constructive Communication	0.40	(0.34, 0.47)	<0.001	0.32	(0.24, 0.39)	<0.001
Mutual Avoidance and Withholding	−0.35	(−0.43, −0.28)	<0.001	−0.25	(−0.32, −0.17)	<0.001
Trust	0.44	(0.37, 0.51)	<0.001	0.29	(0.21, 0.36)	<0.001
Internal Control Index	0.27	(0.20, 0.34)	<0.001	0.23	(0.16, 0.30)	<0.001
Alcohol Dependence	−0.04	(−0.11, 0.03)	0.24	0.02	(−0.05, 0.08)	0.65

Notes: SASE, Score on Sexual Agreement Self-Efficacy scale; ISAC, Score on Importance of Sexual Agreement Communication scale. Correlations were estimated in M*plus* version 8 via full-information maximum likelihood (FIML) estimation with robust cluster-adjusted confidence intervals and *p*-values to account for clustering of participants within dyads. *N* = 776.

## Data Availability

The data analyzed in this publication are available from the principal investigator, C.C.H., on reasonable request.

## Appendix A

Sexual Agreement Self-Efficacy (SASE) scale

When answering the following questions, we want you to think of your current agreement in general even though there may be several specific aspects to your agreement.

Response options:

0 Not at all
1 A little
2 Moderately
3 Very much
4 Extremely

1. How confident are you that you can honor your current agreement?
2. When someone you are attracted to is seducing you, how confident are you that you can honor your current agreement?
3. When you are feeling bad about yourself, how likely is it that you will honor your current agreement?
4. How easy is it for you to keep your current agreement?
5. When you are angry with your partner, how confident are you that you will be able to honor your current agreement?
6. When you are anxious about your relationship, how confident are you that you will be able to honor your current agreement?
7. When your relationship has conflict, how confident are you that you can honor your current agreement?

Scoring: The scale is scored by calculating the mean of the seven responses.

Permission: The SASE scale is in the public domain and freely available to use.

## Appendix B

Importance of Sexual Agreement Communication (ISAC) scale

When answering the following questions, we want you to think of your current agreement in general even though there may be several specific aspects to your agreement.

Response options:

0 Not at all
1 A little
2 Moderately
3 Very much
4 Extremely

1. How important is it to talk to your primary partner about your current agreement?
2. How much do you benefit from talking to your primary partner about your current agreement?
3. How important is it to talk about your current agreement when you are unclear about what it is?
4. How important is it to talk about your current agreement when your primary partner is unclear about what it is?
5. How much do you enjoy talking to your primary partner about your current agreement?

Scoring: The scale is scored by calculating the mean of the five responses.

Permission: The ISAC scale is in the public domain and freely available to use.
